# *Propionibacterium acnes*-derived insoluble immune complexes in sinus macrophages of lymph nodes affected by sarcoidosis

**DOI:** 10.1371/journal.pone.0192408

**Published:** 2018-02-05

**Authors:** Yoshimi Suzuki, Keisuke Uchida, Tamiko Takemura, Masaki Sekine, Tomoki Tamura, Asuka Furukawa, Akira Hebisawa, Yumi Sakakibara, Nobuyasu Awano, Tomonari Amano, Daisuke Kobayashi, Mariko Negi, Tomoya Kakegawa, Yuriko Wada, Takashi Ito, Takashige Suzuki, Takumi Akashi, Yoshinobu Eishi

**Affiliations:** 1 Department of Human Pathology, Graduate School and Faculty of Medicine, Tokyo Medical and Dental University, Bunkyo-ku, Tokyo, Japan; 2 Division of Surgical Pathology, Tokyo Medical and Dental University Hospital, Bunkyo-ku, Tokyo, Japan; 3 Division of Pathology, Japanese Red Cross Medical Center, Shibuya-ku, Tokyo, Japan; 4 Clinical Research Center and Pathology Division, National Hospital Organization Tokyo National Hospital, Kiyose, Tokyo, Japan; 5 Department of Respiratory Medicine, Graduate School and Faculty of Medicine, Tokyo Medical and Dental University, Bunkyo-ku Tokyo, Japan; 6 Clinical Respiratory Medicine, Japanese Red Cross Medical Center, Shibuya-ku, Tokyo, Japan; 7 Division of Pathology, Tokyo Kita Medical Center, Kita-ku, Tokyo, Japan; Universitatsklinikum Freiburg, GERMANY

## Abstract

**Background:**

*Propionibacterium acnes* is thought to be a causative agent of sarcoidosis. Patients with sarcoidosis have circulating immune complexes. We attempted to detect *P*. *acnes*-derived immune complexes in sarcoid lesions.

**Methods:**

We evaluated formalin-fixed and paraffin-embedded lymph node samples from 38 sarcoidosis patients and 90 non-sarcoidosis patients (27 patients with necrotizing lymphadenitis, 28 patients with reactive lymphadenitis, 16 patients with colon cancer, 19 patients with gastric cancer) by immunohistochemistry using anti-human immunoglobulins (IgG, IgA, and IgM) and complement (C1q and C3c) antibodies, and a *P*. *acnes*-specific monoclonal antibody (PAB antibody) that reacts with the membrane-bound lipoteichoic acid of *P*. *acnes*.

**Results:**

Small round bodies (SRBs) bound to IgA, IgM, or IgG were detected in sinus macrophages, in 32 (84%), 32 (84%), or 11 (29%) sarcoid samples, respectively, and in 19 (21%), 26 (29%), or no (0%) control samples, respectively. Some of these insoluble immune complexes (IICs) also bound to C1q and C3c. We developed a microwave treatment followed by brief trypsin digestion (MT treatment) to detect PAB-reactive SRBs bound to immunoglobulins (IIC-forming *P*. *acnes*). MT treatment revealed abundant IIC-forming *P*. *acnes* in most (89%) of the sarcoid samples and sparse distribution in some (20%) of the control samples with lymphadenitis, but no IIC-forming *P*. *acnes* was detected in control samples without inflammation. IIC-forming *P*. *acnes* were mostly bound to both IgA and IgM. The PAB-reactive antigen and immunoglobulins were both located at the peripheral rim of the IIC-forming *P*. *acnes*. Conventional electron microscopy identified many SRBs (0.5–2.0 μm diameter) in sinus macrophages of sarcoid lymph nodes with many IIC-forming *P*. *acnes*, some of which were in phagolysosomes with a degraded and lamellar appearance.

**Conclusions:**

*P*. *acnes*-derived IICs in sinus macrophages were frequent and abundant in sarcoid lymph nodes, suggesting a potential etiologic link between sarcoidosis and this commensal bacterium.

## Introduction

Sarcoidosis, a systemic granulomatous disease involving multiple organs, has an unknown etiology. Recent studies, however, have implicated *Propionibacterium acnes* as a causative agent of sarcoidosis [1,2]. In bacterial culture, *P*. *acnes* cells were isolated from 78% of sarcoid lymph nodes and 21% of control lymph nodes [3]. Quantitative polymerase chain reaction detected high amounts of *P*. *acnes* DNA in 80% of sarcoid lymph nodes and some *P*. *acnes* DNA in 15% of control lymph nodes [4]. *In situ* hybridization with signal amplification by catalyzed reporter deposition [5] and immunohistochemistry (IHC) with a novel monoclonal antibody (PAB) that reacts with *P*. *acnes* lipoteichoic acid (PLTA) detected this commensal bacterium in granuloma cells of sarcoid lymph nodes [6]. Th1 immune responses to *P*. *acnes* are increased in sarcoidosis patients [7–11]. Sarcoid granulomas may thus be caused by *P*. *acnes* in susceptible subjects with Th1 hypersensitivity to the commensal bacterium [1].

Circulating immune complexes are reported in sarcoidosis patients with the proportion varying between 3% and 58% depending on the detection technique used [12–17]. Insoluble immune complexes (IICs) in sarcoid lymph nodes were first described at the XI World Congress of Sarcoidosis and Other Granulomatous Disorders held in Milan, Italy, in 1988 [18]. The etiology and role of these immune complexes in sarcoidosis, however, remained uncertain for a long time. Epithelioid cell granuloma formation is basically a host defense mechanism against strongly antigenic and poorly degradable substances, including microorganisms. Such antigenic and insoluble substances form IICs in sensitized hosts, which enhances granulomatous inflammation and granuloma formation [19]. To evaluate the *P*. *acnes* etiology of sarcoidosis, we sought to detect IICs that were formed against *P*. *acnes* (i.e., IIC-forming *P*. *acnes*) in sarcoid lymph nodes.

## Materials and methods

### Samples

Lymph node samples were obtained from 38 sarcoidosis patients and 90 non-sarcoidosis patients, including 27 patients with necrotizing lymphadenitis, 28 patients with reactive lymphadenitis, 16 patients with colon cancer, and 19 patients with gastric cancer, between April 1995 and March 2017, at the Tokyo Medical and Dental University Hospital, Japanese Red Cross Medical Center, National Hospital Organization Tokyo National Hospital, and Tokyo Kita Medical Center. These lymph node samples were 10% neutral buffered formalin-fixed and paraffin-embedded (FFPE) in the pathology laboratories of each hospital. The diagnosis of sarcoidosis was based on both histologic and clinical findings according to the statement of the American Thoracic Society/European Respiratory Society/World Association of Sarcoidosis and other Granulomatous Disorders on sarcoidosis [20]. Clinical profiles of the sarcoidosis patients from which the lymph node samples were obtained are shown in Table 1. The diagnosis of necrotizing and reactive lymphadenitis as a control was established at each hospital and histologically confirmed before the study. Samples from cancer patients were all draining lymph nodes without metastasis from the primary cancer. The clinical profiles of the control patients are shown in S1 Table. No differences were detected between the sarcoidosis and control patients in terms of age, sex, and concentration of serum immunoglobulins, although the mean age of the sarcoidosis patients was greater than that of control patients with reactive or necrotizing lymphadenitis and lower than that of control patients with gastric or colon cancer. The ethics committee of each hospital approved our study: Tokyo Medical and Dental University Hospital (reference M2000-2218, September 27, 2015), Japanese Red Cross Medical Center (reference 625, October 21, 2015), National Hospital Organization National Hospital (reference 313, April 27, 2016); and Tokyo Kita Medical Center (reference 173, March 8, 2017). The need for consent was waived by these ethics committees.

**Table 1:** Clinical profiles of patients with sarcoidosis.

Clinical Characteristics	
Number	38
Subjects (men / women)	16 / 22
Age, years	56.5 ± 16.2
Smoking history	
Never Smoker	16
Current Smoker	13
Ex-smoker	2
NA ^a^	7
CT findings (positive/negative) ^b^	
BHL ^c^	31/7
Mediastinal nodal enlargement	27/11
Lung parenchymal disease	20/18
Chest X-ray Stage (0/I/II/III/IV)	7/11/13/2/5
Extra-thoracic lesions	
Eye	12
Nerve	3
Lymph node	2
Heart	1
Liver	1
Muscle	1
Skin	1
Location of lymph node biopsy	
Hilum	9
Mediastinal	6
Tracheal	2
Abdominal	1
Para arterial	1
Cervical	8
Inguinal	4
Supraclavicular	4
Anterior scalen	3
ACE (U/I/37°C) ^d^	18.1± 8.6
Lysozyme (μg/ml) ^e^	11.4 ± 6.1
sIL-2R (U/ml) ^f^	1594± 1373
Serum Immunoglobulin ^g^	
IgG (mg/dl)	1484.1 ± 448.7
IgA (mg/dl)	293.5 ± 134.4
IgM (mg/dl)	102.5 ± 45.9
BAL analysis ^h^	
CD4/CD8 ratio	5.1 ± 4.9

^a^ NA: not available,

^b^ CT: computed tomography,

^c^ BHL: bilateral hilar lymphadenopathy,

^d^ ACE: angiotensin converting enzyme,

^f^ sIL-2R: soluble interleukin-2 receptor, and

^h^ BAL: bronchoalveolar lavage.

Normal range: ^d^ 8.3–21.4, ^e^ 5.0–10.2, ^f^ < 710, ^g^ IgG; 1009–1338, IgA; 150–203, IgM; 102–131, and ^h^ CD4/CD8 ratio; <3.5.

### Enzyme immunohistochemistry

Histologic sections (3 μm-thick) cut from FFPE lymph node samples were mounted on silane-coated slides (Muto Pure Chemicals Co. Ltd., Tokyo, Japan), de-paraffinized, and rehydrated, and then pretreated with the appropriate antigen retrieval methods for each antigen.

To detect human immunoglobulins and complement, the sections were incubated in phosphate-buffered saline (PBS pH7.2) containing 0.25% trypsin (DIFCO Laboratories, Detroit, MI, USA) for 30 min at 37°C. To detect *P*. *acnes*, the sections were microwaved (Microwave Processor H2850; Energy Beam Sciences, East Granby, CT, USA) in 10 mmol/l citrate buffer (pH 6.0) for 40 min at 97°C, according to the previously described method [6]. To detect IIC-forming *P*. *acnes*, we developed a novel method (MT treatment) to expose the IIC core antigens. For the MT treatment, sections were microwaved in 10 mmol/l citrate buffer (pH 6.0) for 5 min at 97°C, and then incubated in PBS containing 0.25% trypsin (DIFCO) at 37°C for 10 s.

After performing the antigen retrieval procedures described above, we first incubated the sections for 10 min in 3% hydrogen peroxide and methanol, and then with normal horse serum (Vectastain Universal Elite ABC Kit; Vector Laboratories, Burlingame, CA, USA) followed by overnight incubation at room temperature in the appropriately diluted primary antibody. Rabbit anti-human IgG, IgA, and IgM antibodies were ready-to-use products obtained from DAKO (IR512, 510, and 513, respectively, Glostrup, Denmark). Rabbit anti-human C1q and C3c were also purchased from DAKO (0136 and A062, respectively) and diluted 1:20,000 and 1:6000, respectively. The mouse anti-*P*. *acnes* monoclonal antibody (PAB: IgM, κ) specific to PLTA was diluted 1:60,000 and used as a primary antibody to detect *P*. *acnes* or IIC-forming *P*. *acnes*. A mouse anti-mycobacterium monoclonal antibody (LAM antibody: IgM, κ) specific to lipoarabinomannan [21] or PBS only was used as the primary antibody in controls. After reacting with each primary antibody, we incubated the sections at room temperature with biotinylated secondary antibody for 30 min and then with streptavidin–peroxidase complex for another 30 min (Vectastain Universal Elite ABC Kit). The sections were washed before and after each step in PBS containing 0.5% Tween-20 (T-PBS). Diaminobenzidine (Histofine Simplestain DAB Solution; Nichirei Bioscience, Tokyo, Japan) was used as the chromogen to produce a brown reaction product. All specimens were counterstained with Mayer’s hematoxylin. Hematoxylin and eosin staining was used on adjacent sections for further histologic examination.

The specificity of the PAB antibody for *P*. *acnes* was evaluated by IHC with the PAB antibody with or without MT treatment using FFPE rat liver sections obtained after intravenously injecting 30 mg of various species of heat-killed bacteria (S2 Table) into female Sprague–Dawley rats (CLEA Japan Inc.) 1 h before killing the rats. The specificity of the PAB antibody in the IHC with MT treatment was also evaluated by in situ hybridization (ISH) using catalyzed reporter deposition for signal amplification with a digoxigenin-labeled oligonucleotide probe that complemented the 16S rRNA of *P*. *acnes* using the previously described method [5].

### PAB-reactivity blocking experiments with human plasma samples

PAB-antibody reactivity blocking experiments were performed using human plasma samples from three sarcoidosis patients and three healthy adult volunteers with enzyme-linked immunosorbent assay (ELISA) plates coated with PLTA, as well as with FFPE tissue sections from sarcoid lymph node samples with many IIC-forming *P*. *acnes*, and from *P*. *acnes*-infected rat liver obtained by intravenously injecting heat-killed *P*. *acnes* (ATCC 6919), as described above.

For the ELISA, the PLTA was purified from *P*. *acnes* (ATCC 6919) as previously described [6] with modifications. Flat-bottomed 96-well NUNC-immuno plates (Nalge Nunc International, Roskilde, Denmark) were coated with PLTA (500 ng/well) in carbonate-bicarbonate buffer (pH 9.6) for 60 min at 37°C. Human plasma (final dilution 1:200 in PBS) or PBS as a control was added to each well and the plates were incubated for 60 min at 37°C. After the initial incubation, the plates were further incubated for 60 min at 37°C with unlabeled mouse anti-PLTA antibody (PAB) diluted 1:500; unlabeled rabbit anti-human C1q and C3c (DAKO 0136 and A062, respectively) diluted 1:500; or biotinylated goat anti-human IgG, IgA, or IgM antibody (Invitrogen, Carlsbad, CA, USA) diluted 1:5000. For the biotinylated antibodies, the plates were incubated for 30 min at room temperature with horseradish peroxidase-conjugated streptavidin (P0397, DAKO), and for the unlabeled antibodies, the plates were incubated for 30 min with biotinylated anti-mouse immunoglobulins (E0354, DAKO) diluted 1:5000 or biotinylated anti-rabbit immunoglobulins (E0353, DAKO) diluted 1:5000, and then incubated for 30 min with horseradish peroxidase-conjugated streptavidin (P0397, DAKO) diluted 1:5000 at room temperature. The plates were washed with T-PBS before and after each step. Citrate phosphate buffer (pH 5.4) containing 0.3% o-phenylenediamine dihydrochloride (Sigma-Aldrich, St. Louis, MO, USA) and 0.012% H_2_O_2_ were then added to each well, and the plates were incubated in the dark at room temperature for 15 min. To stop the reaction, we added 25 μl of 2 N HCl to each well, and the plates were read at 490 nm on a Bio-Kinetics Reader (Bio-Tek Instruments Inc., VT, USA). Assay results were determined as the mean of three identical wells from each sample.

For tissue blocking experiments, enzyme IHC was performed according to the method for detecting IIC-forming *P*. *acnes* described above with some modifications. Before the reaction with the PAB antibody as the primary antibody, the sections were incubated with undiluted human plasma samples or PBS as a control for 2 h at room temperature and then washed in T-PBS, followed by 30-min fixation with 10% neutral buffered formalin, which is commonly used for routine pathologic examination. After washing in T-PBS, enzyme IHC of the sections was continued after incubation with the PAB antibody, as described above. These blocking experiments were similarly performed using immunoglobulin fractions (separated by 50% ammonium sulfate saturation or Protein G-sepharose [29048581, GE Healthcare UK, Little Chalfont, UK]) of a plasma sample from one of the three healthy volunteers.

### Double fluorescence immunohistochemistry

Three sarcoid samples containing many IICs were evaluated by immunofluorescence double-staining to evaluate the co-localization of PLTA with IgA, IgM, and C3c. Double-staining of PLTA and IgA, IgM, or C3c was performed by incubating tissue sections (3 μm-thick) with normal horse serum after MT treatment, and then with the PAB antibody for 60 min at room temperature. The tissue sections were subsequently reacted with a biotinylated secondary antibody (Vectastain Universal Elite ABC Kit) followed by 30-min incubation in the dark with fluorescein isothiocyanate-conjugated streptavidin (F0250, DAKO) diluted 1:50, both at room temperature. For IgA and IgM double-staining, the sections were microwaved for 40 min at 97°C, incubated with normal horse serum, and then further incubated for 60 min at room temperature with the anti-human IgA (IR510, DAKO), followed by the fluorescence protocol after the primary antibody reaction, as described above. After the first reaction with the PAB antibody or anti-IgA antibody, the sections were microwaved again for 20 min and the second reaction was the same for all of the double-staining combinations: the sections were incubated for 60 min at room temperature with anti-human IgA, IgM, or C3c, followed by 30-min incubation in the dark with tetramethylrhodamine isothiocyanate-conjugated anti-rabbit immunoglobulin (R0150, DAKO) diluted 1:50 at room temperature. The sections were washed in T-PBS before and after each step, and coverslipped using fluorescence mounting medium (DAKO). A fluorescence laser-scanning microscope (FV1200, Olympus, Tokyo, Japan) was used to examine and photograph the stained sections.

### Immuno-electron microscopy

Immuno-electron microscopy was used to examine the same samples evaluated by fluorescence double-staining. Paraffin sections (6 μm-thick) were prepared as for enzyme IHC up to the secondary antibody reaction step, fixed with 2.5% glutaraldehyde for 5 min on ice, and incubated with peroxidase substrate (Histofine Simplestain DAB Solution). The sections were washed with PBS several times, post-fixed with 0.5% OsO_4_ for 60 min, washed again with PBS, and then dehydrated in an ascending series of ethanol and embedded in Epon 812 (TAAB Laboratories Ltd. Berks, England). For flat embedding, gelatin capsules filled with Epon 812 were positioned on top of section areas that were selected based on parallel sections treated for enzyme IHC. After polymerization, the Epon blocks containing the tissue were peeled from the glass slides by heating, and then trimmed precisely with small tissue areas (1 mm^2^) readily identifiable on the block surface under reflective light. Ultrathin sections were cut using a Reichert Ultracut S (Leica EM UC7 Microsystems Heidelberg GmbH, Mannheim, Germany) and collected on Maxtaform grids (Pyser-SGL Ltd., Kent, UK). Sections were then stained with lead citrate and examined under an H-7700 electron microscope (Hitachi High-Technologies Co., Tokyo, Japan). Two sarcoid lymph node samples containing many IICs available for conventional electron microscopy were used to identify the SRBs corresponding to PAB-reactive IICs observed by enzyme IHC and immune-electron microscopy.

### Statistical analyses

A one-way analysis of variance (ANOVA) and further analysis using the Holm-Sidak multiple comparisons test was used to evaluate differences in the clinical profiles between the sarcoidosis and control groups. Fisher’s exact test was used to evaluate differences in enzyme IHC detection frequency between the sarcoidosis and control groups. The analyses were performed with GraphPAD PRISM ver. 6 (GraphPad Software, Inc., San Diego, CA, USA). *P* < 0.05 was considered statistically significant.

## Results

### Immunoglobulin-bound SRBs in sinus macrophages

**Fig 1:**
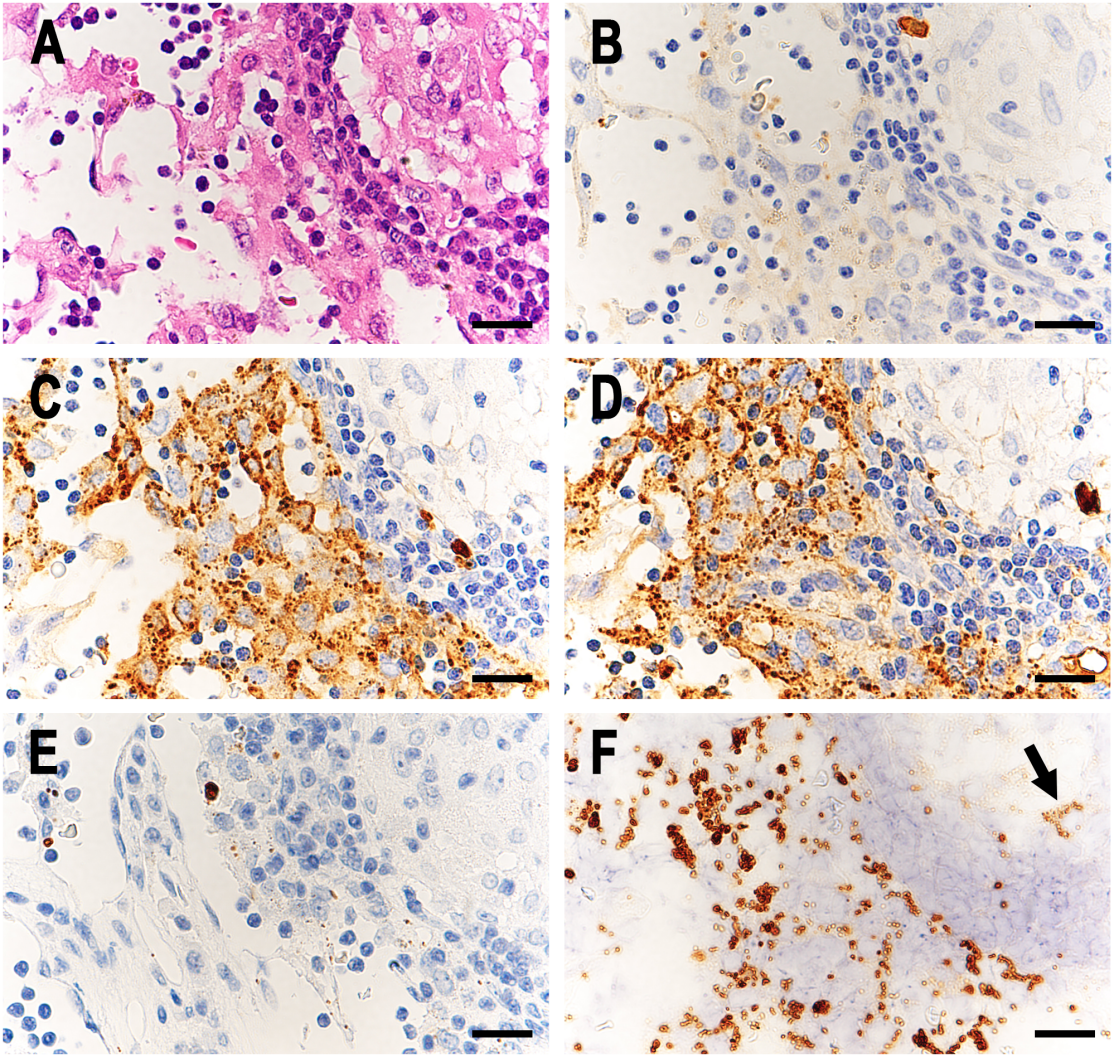
*P*. *acnes*-derived insoluble immune complexes in sinus macrophages of sarcoid lymph nodes. In a representative case of sarcoid lymph nodes, identical areas of the lesion including a lymphatic sinus and adjacent paracortical area with a sarcoid granuloma are shown in semi-serial sections; HE stain (A), IHC with anti-human IgG (B), IgA (C), and IgM (D) antibody, IHC with PAB antibody (E), and IHC with PAB antibody after MT treatment (F). In the sinus macrophages, the distributions of IgA- and IgM-positive SRBs (C and D) and PAB-reactive SRBs (F) were similar. Note the few PAB-reactive SRBs with weak intensity (indicated by the arrow) in the granuloma after MT treatment (F). Scale bar: 20 μm.

**Fig 2:**
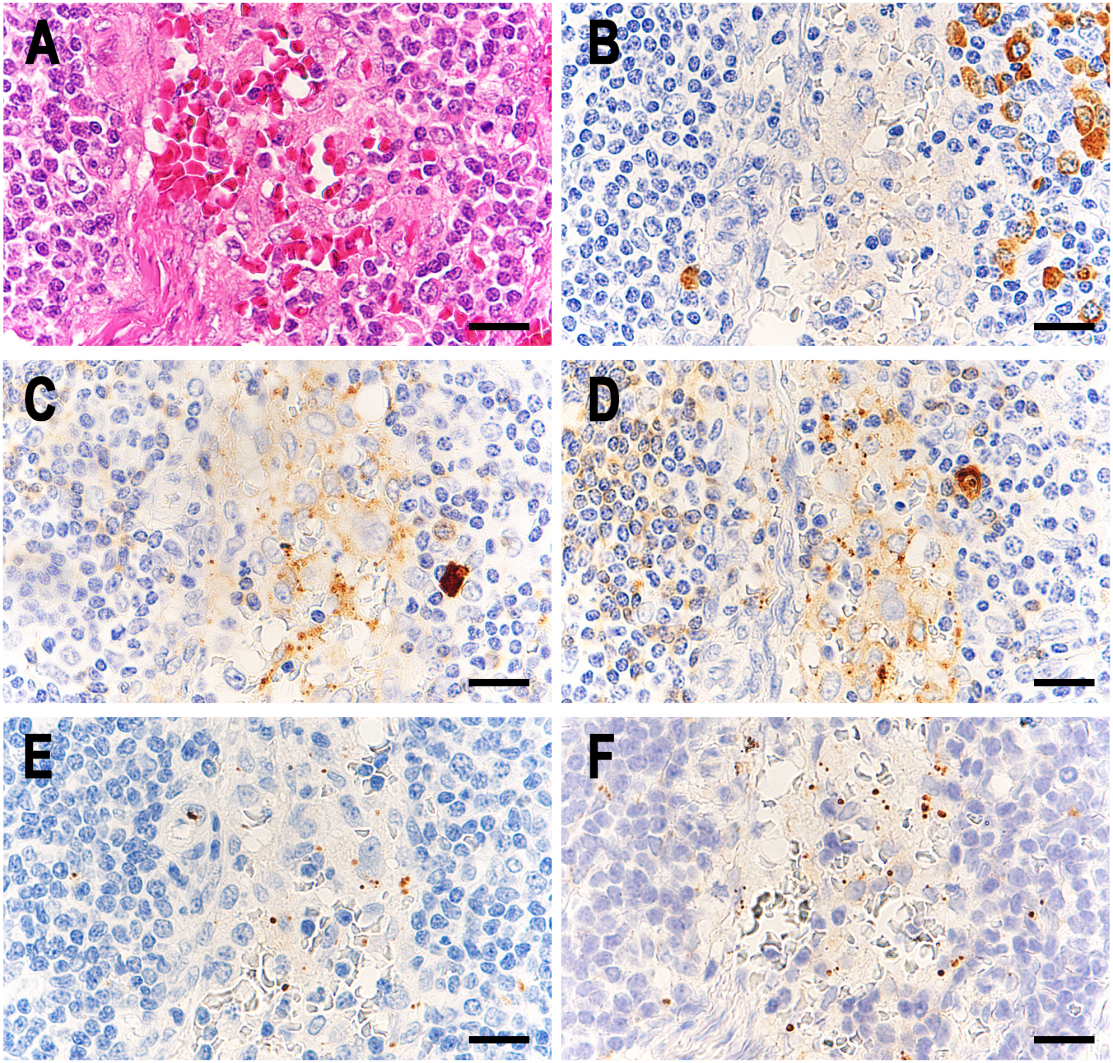
Insoluble immune complexes in sinus macrophages of control lymph nodes from patients with reactive lymphadenitis. In a representative case of control lymph nodes with IICs from patients with reactive lymphadenitis, identical areas of the lymphatic sinus are shown in semi-serial sections; HE stain (A), IHC with anti-human IgG (B), IgA (C), and IgM (D) antibody, IHC with PAB antibody (E), and IHC with PAB antibody after MT treatment (F). In the sinus macrophages, IgA- and IgM-positive SRBs were detected (C and D, respectively), and PAB-reactive SRBs were also detected with a small increase in the number after MT treatment (F). Scale bar: 20 μm.

**Fig 3:**
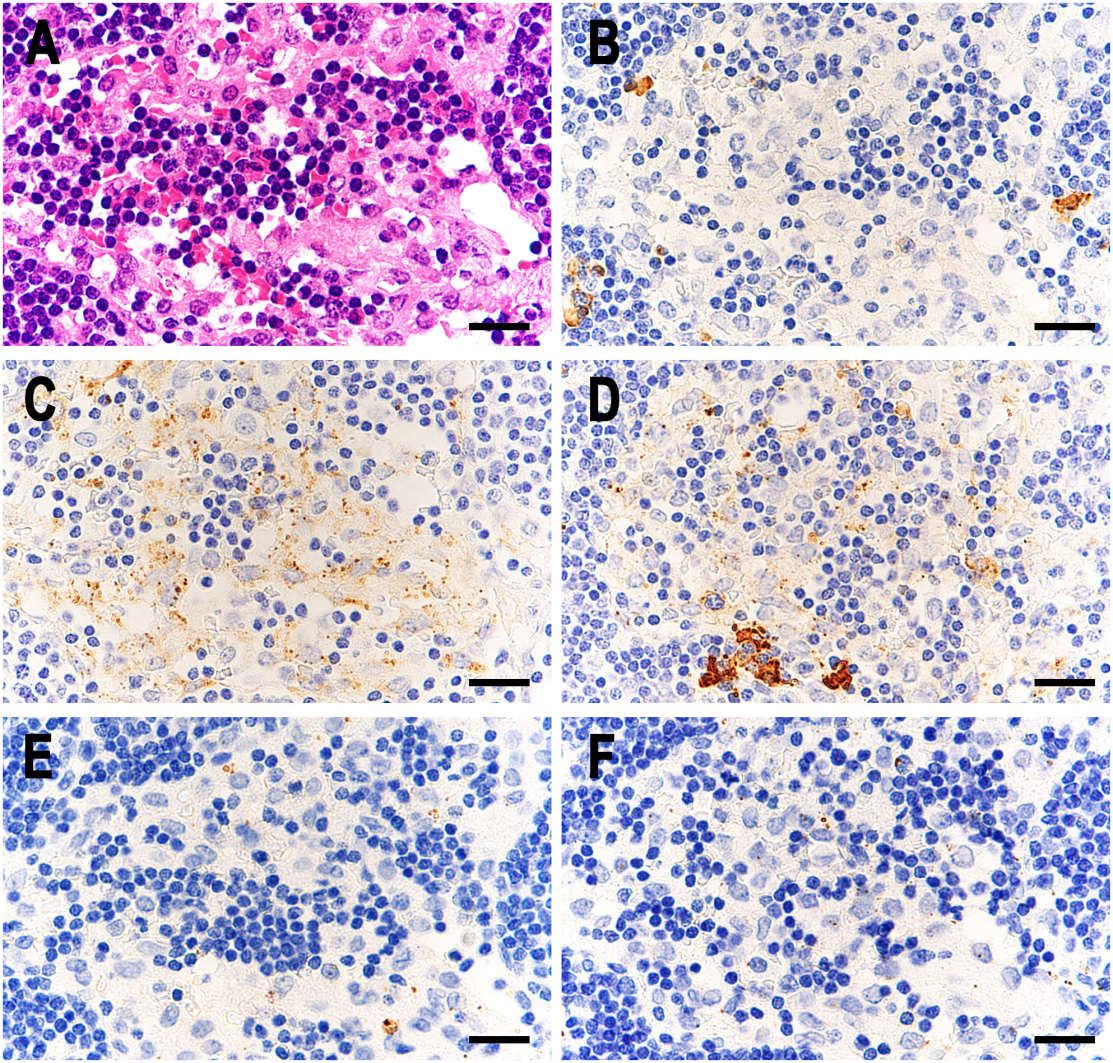
Insoluble immune complexes in sinus macrophages of control lymph nodes from colon cancer patients. In a representative case of control lymph nodes with IICs from colon cancer patients, identical areas of the lymphatic sinus are shown in semi-serial sections; HE stain (A), IHC with anti-human IgG (B), IgA (C), and IgM (D) antibody, IHC with PAB antibody (E), and IHC with PAB antibody after MT treatment (F). In the sinus macrophages, many IgA-positive and a few IgM-positive small particles were detected (C and D, respectively), although a few PAB-reactive SRBs were detected with no difference in the number between the sections with and without MT treatment (F and E, respectively). Scale bar: 20 μm.

**Fig 4:**
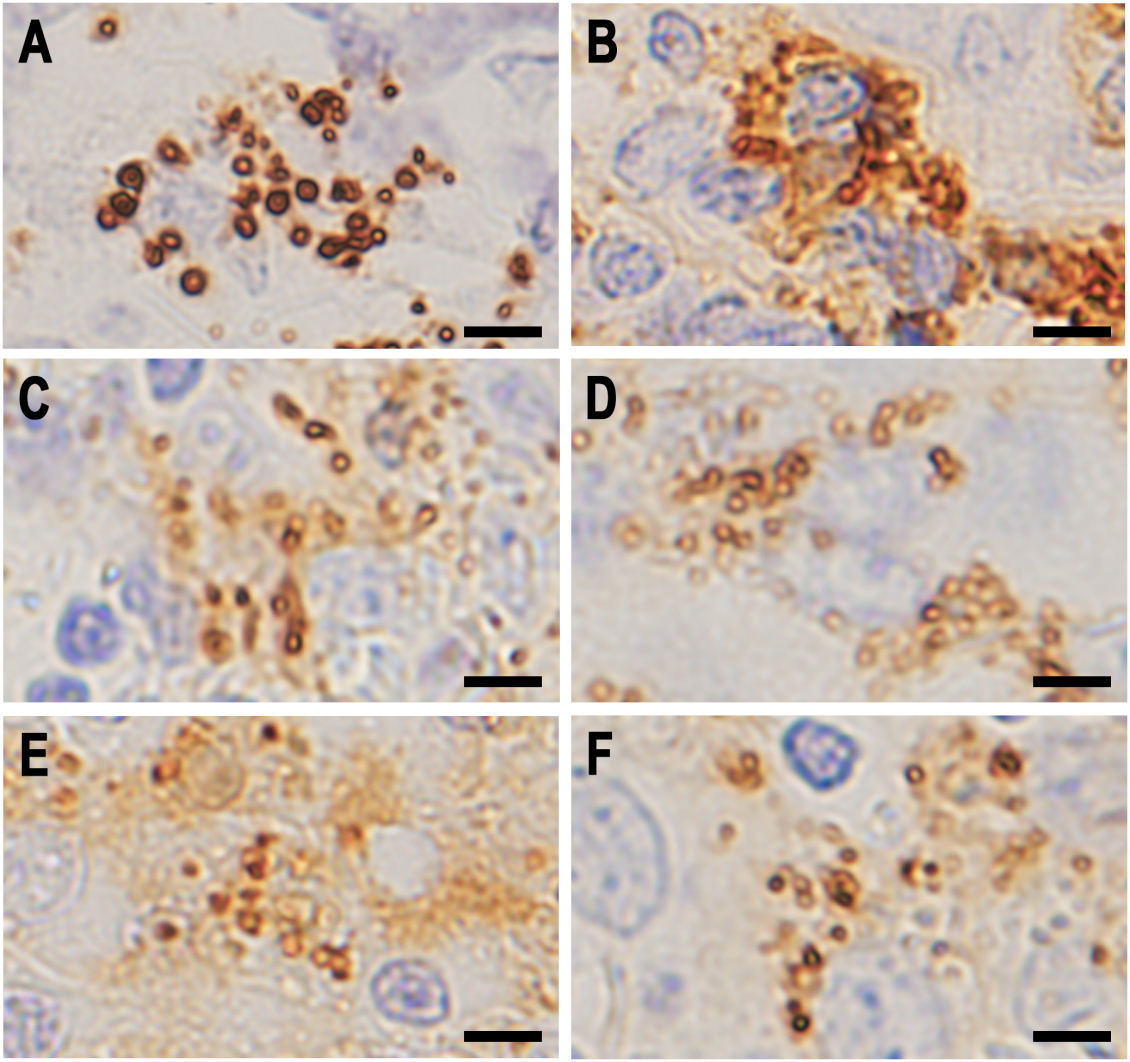
Higher magnification of small round bodies detected in the lymphatic sinus of sarcoid lymph nodes by IHC with each antibody. A: IHC with PAB antibody after MT treatment, B: IHC with anti-IgG antibody, C: IHC with anti-IgA antibody, D: IHC with anti-IgM antibody, E: IHC with anti-C1q antibody, and F: IHC with anti-C3c antibody. Note that the dark brown-colored reaction products produced by each antibody are all located along the peripheral rim of the small round bodies. Scale bar: 5 μm.

SRBs bound to immunoglobulins were detected in many sinus macrophages of many sarcoid samples (Fig 1) and in a few sinus macrophages of some control samples (Figs 2 and 3). Complement was also detected in some of these immunoglobulin-bound SRBs. Most of the Hamazaki-Wesenberg (HW) bodies were negative for immunoglobulins and complement. High-power magnification of IHC sections showed that immunoglobulins and complement were located at the peripheral margin of the SRBs in sinus macrophages (Fig 4). The detection frequency of SRBs bound to immunoglobulins (IgG, IgA, or IgM) and complement (C1q or C3c) is shown in Table 2. The frequency of IgA-bound or IgM-bound SRBs was higher in sarcoid samples than in control samples. Many (76%) sarcoid samples contained both IgA-bound and IgM-bound SRBs. IgG-bound SRBs were detected in some sarcoid samples and in none of the control samples. Sarcoid samples containing IgG-bound SRBs also contained both IgA-bound and IgM-bound SRBs. C1q-bound or C3c-bound SRBs were detected in some sarcoid and control samples with IgA-bound and/or IgM-bound SRBs.

**Table 2:** Frequency of insoluble immune complexes positive for IgG, IgA, IgM, C1q, or C3c in sinus macrophages of sarcoid and control lymph nodes.

Patients with	n	Number (%) of samples with insoluble immune complexes positive for				
		IgG	IgA	IgM	C1q	C3c
**Sarcoidosis**	**38**	**11 (29%)**	**32 (84%)**	**32 (84%)**	**11 (29%)**	**3 (8%)**
**Control diseases**	**90**	**0** *^1^	**19 (21%)** *^1^	**26 (29%)** *^1^	**9 (10%)** *^8^	**4 (4%)**
Colon cancer	16	0 *^2^	3 (19%) *^1^	4 (25%) *^1^	0 *^9^	0
Gastric cancer	19	0 *^3^	5 (26%) *^1^	7 (37%) *^6^	1 (5%) *^10^	1 (5%)
Necrotizing lymphadenitis	27	0 *^4^	3 (11%) *^1^	4 (15%) *^1^	1 (4%) *^11^	1 (4%)
Reactive lymphadenitis	28	0 *^5^	8 (29%) *^1^	11 (39%) *^7^	7 (25%)	2 (7%)

*^1^; *P* < 0.0001,

*^2^; *P* = 0.0226,

*^3^; *P* = 0.0103,

*^4^; *P* = 0.0017,

*^5^; *P* = 0.0016,

*^6^; *P* = 0.0006,

*^7^; *P* = 0.0002,

*^8^; *P* = 0.0142,

*^9^; *P* = 0.0226,

*^10^; *P* = 0.0452, and

*^11^; *P* = 0.0103 compared with sarcoidosis patients (Fisher's exact test).

### PAB-reactive SRBs in sinus macrophages

A few PAB-reactive SRBs were detected by IHC without MT treatment and many PAB-reactive SRBs were detected by IHC with MT treatment in the sinus macrophages of sarcoid lymph nodes (Figs 1 and 4). No positive signal was observed by IHC with or without MT treatment when the LAM antibody or PBS was used instead of the PAB antibody (S1 Fig). MT treatment did not affect the specificity of the PAB antibody to *P*. *acnes* (S2 Table). The distribution of ISH-positive signals for *P*. *acnes* DNA was almost identical to that of the IHC-positive signals obtained with the PAB antibody after MT treatment (S2 Fig). The frequency and location of PAB-reactivity in the sarcoid and control lymph node samples with or without MT treatment are shown in Table 3. After MT treatment, not only the frequency, but also the number of PAB-reactive SRBs in sinus macrophages was markedly increased in many sarcoid samples (Fig 1E and 1F), whereas in the control samples with lymphadenitis, the MT treatment-induced increase was much smaller (Fig 2). MT treatment did not change the frequency or the number of PAB-reactive SRBs in non-inflammatory control lymph nodes from cancer patients (Fig 3). The detection status of PAB-reactive SRBs and immunoglobulin-bound SRBs in sinus macrophages did not correlate with the clinical profiles of sarcoidosis and control patients, including the location of lymph nodes obtained by biopsy. After MT treatment, the number of PAB-reactive SRBs in granuloma cells was increased in some sarcoid samples (S3 Fig), and the frequency was also slightly increased. PAB-reactive HW bodies in sinus macrophages were observed in both sarcoid and control samples with higher frequency in sarcoid samples. MT treatment did not affect the PAB-reactivity of the HW bodies.

**Table 3:** Frequency of PAB-reactive granuloma cells, HW-bodies, and SRBs in sarcoid and control lymph nodes.

Patients with	n	Number (%) of samples with PAB-reactivity (without MT treatment)			Number (%) of samples with PAB-reactivity (with MT treatment)		
		granuloma cell	HW body	small round body	granuloma cell	HW body	small round body
**Sarcoidosis**	**38**	**35 (92%)**	**26 (68%)**	**6 (16%)**	**36 (95%)**	**26 (68%)**	**34 (89%)**
**Control diseases**	**90**	-	**8 (9%)** *	**6 (7%)**	-	**8 (9%)** *	**15 (17%)** *
Colon cancer	16	-	7 (44%)	2 (13%)	-	7 (44%)	2 (13%) *
Gastric cancer	19	-	1 (5%) *	2 (11%)	-	1 (5%) *	2 (11%) *
Necrotizing lymphadenitis	27	-	0 *	1 (4%)	-	0 *	4 (15%) *
Reactive lymphadenitis	28	-	0 *	1 (4%)	-	0 *	7 (25%) *

* *P* < 0.0001 compared with sarcoidosis patients (Fisher's exact test).

### PAB-reactivity blocking experiments with human plasma samples

**Fig 5:**
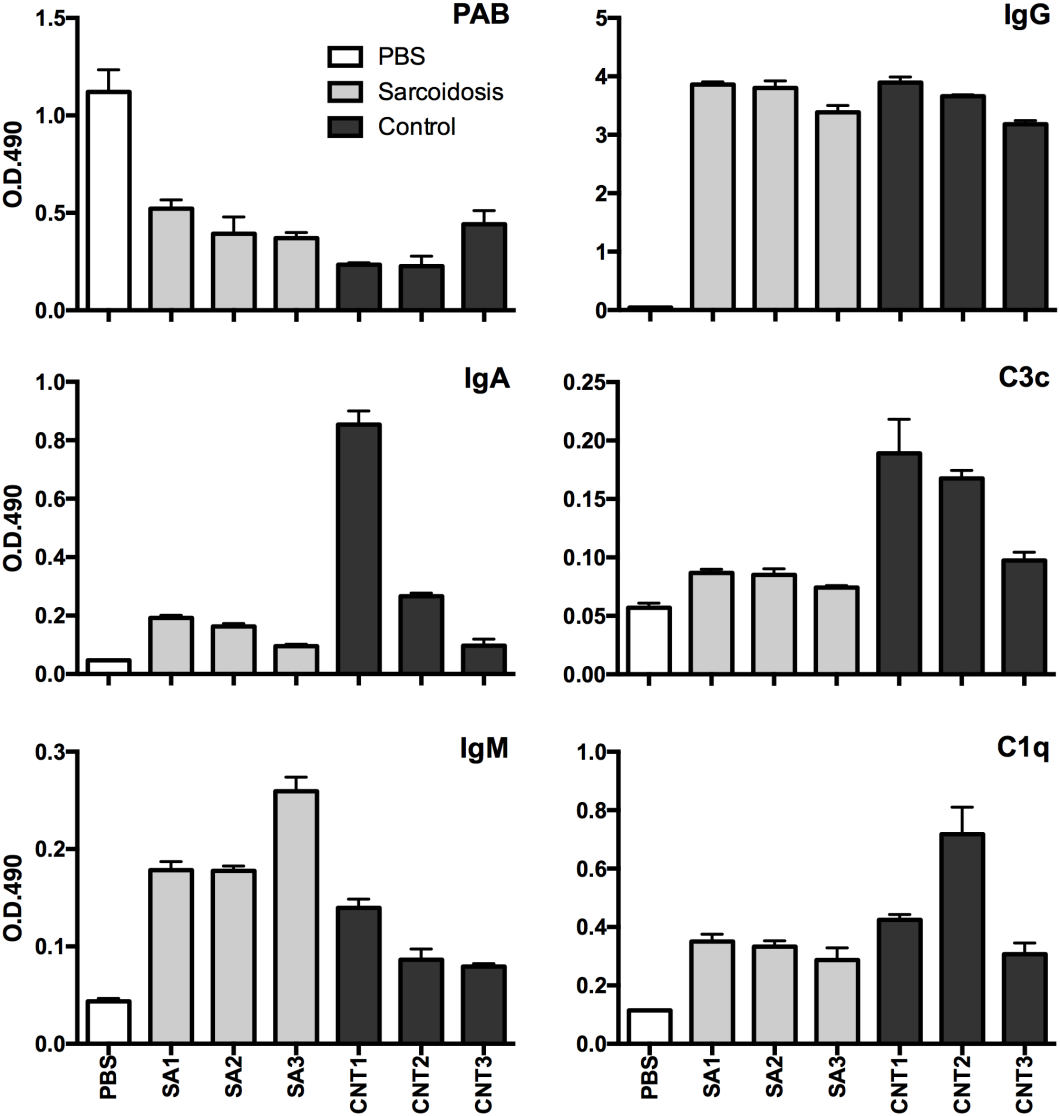
Inhibition of PAB-reactivity with human plasma samples and simultaneous binding of immunoglobulins and complement on PLTA-coated ELISA plates. The ELISA plates coated with PLTA were first incubated with PBS or human plasma samples from three sarcoidosis patients (SA1-3) or three healthy adult volunteers (CNT1-3). After incubation, PAB-reactivity and binding of immunoglobulins and complement with PLTA were measured by ELISA. Assay results (O.D. 490 nm) were determined as the mean of three identical wells.

**Fig 6:**
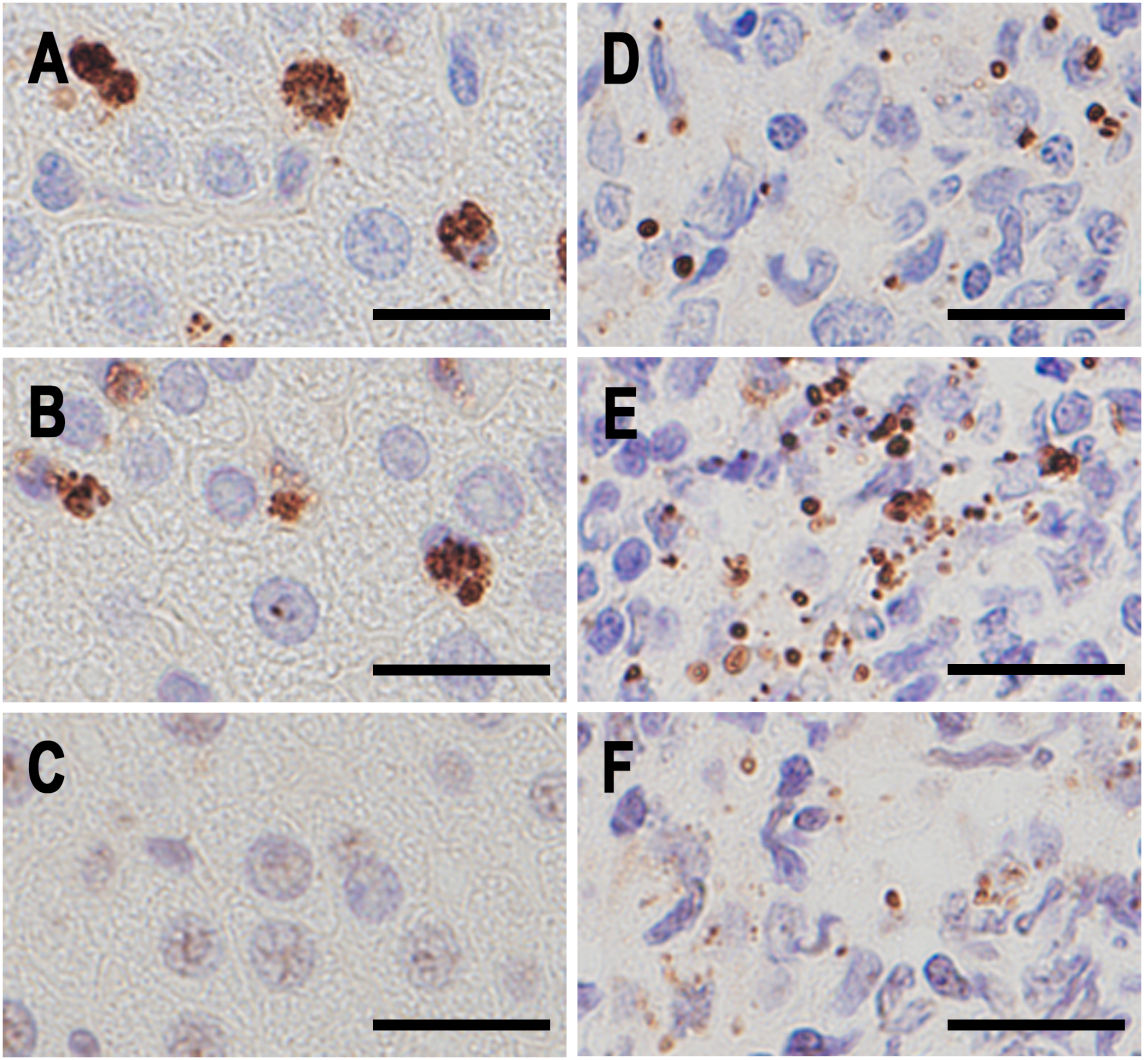
PAB-reactivity blocking experiment in tissue sections of P. acnes-infected rat liver and a sarcoid lymph node containing many IICs. Identical areas of serial sections from *P*. *acnes*-infected rat liver (A-C) and from a sarcoid lymph node containing many IICs in sinus macrophages (D-F). A and D: IHC with the PAB antibody, B and E: IHC with the PAB antibody after MT treatment, and C and F: IHC with the PAB antibody after MT treatment and subsequent reaction with a human plasma sample from a healthy adult volunteer. Scale bar: 20 μm.

**Fig 7:**
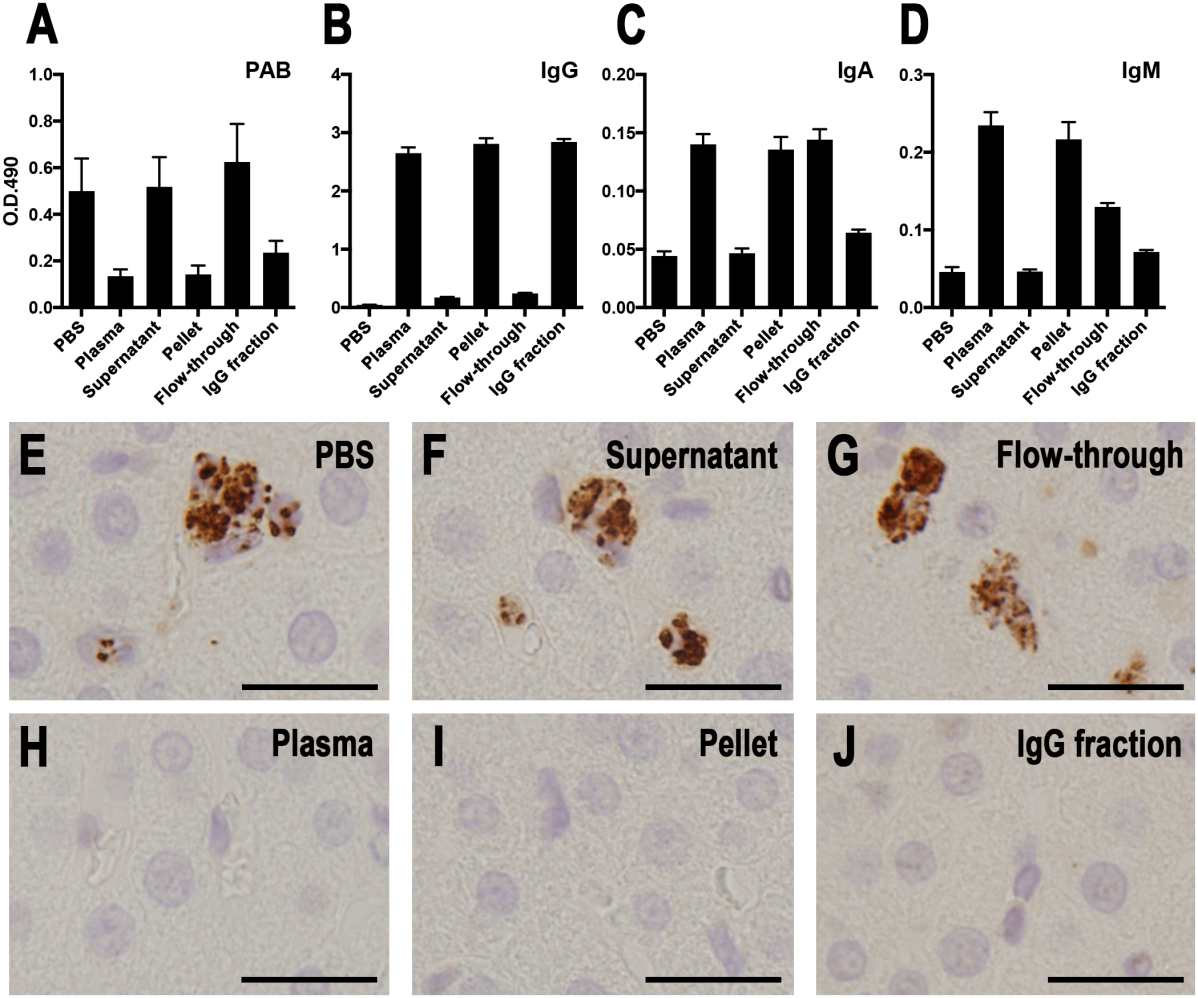
PAB-reactivity blocking experiments with fractionated human plasma samples by 50% ammonium sulfate saturation and Protein G-sepharose. PAB antibody-reactivity blocking experiments with fractionated plasma samples were performed using ELISA coated with PLTA (A-D) and IHC on histologic sections of the *P*. *acnes*-infected rat liver (E-J). A plasma sample from a healthy adult volunteer was fractionated by 50% ammonium sulfate saturation (pellet and supernatant) and Protein G-sepharose (IgG fraction and flow-through). These blocking experiments were performed according to the procedures described in the Materials and Methods. The results of the ELISA (O.D. 490 nm) are shown as the mean of 12 identical wells from each sample. The IHC results are shown in correspondence with each sample tested, including PBS (E), original plasma sample (H), supernatant (F), pellet (I), flow-through (G), and the IgG fraction (J). Scale bar: 20 μm.

To examine possible epitope competition between the PAB antibody used for IHC and anti-PLTA antibodies in human plasma samples, we performed blocking experiments using ELISA and IHC. On the ELISA plates coated with PLTA, diluted (1:200) human plasma samples applied before the reaction interfered with subsequent PAB antibody binding, regardless of the source of the plasma samples (Fig 5). After incubating with human plasma samples, the PLTA coated on the ELISA plates bound to plasma IgG-, IgM-, IgA- antibodies coupled with complement, C1q and C3c, with varying degrees of reactivity among the samples. On the *P*. *acnes*-infected rat liver histologic sections, application of undiluted human plasma samples before the reaction completely abolished positive reaction signals with the bacteria in Kupffer cells by IHC using the PAB antibody (Fig 6A–6C). On the sarcoid lymph node histologic sections, the increase in the number of PAB-reactive SRBs in sinus macrophages after MT treatment was blocked by first applying undiluted human plasma samples (Fig 6D–6F). The ability of plasma samples to block PAB-reactivity with PLTA on ELISA plates or *P*. *acnes* on tissue sections was sustained when the blocking experiments were performed using the immunoglobulin fraction separated by ammonium sulfate or the IgG fraction separated by Protein G (Fig 7).

### Identical localization of PAB-reactivity and immunoglobulins in SRBs

**Fig 8:**
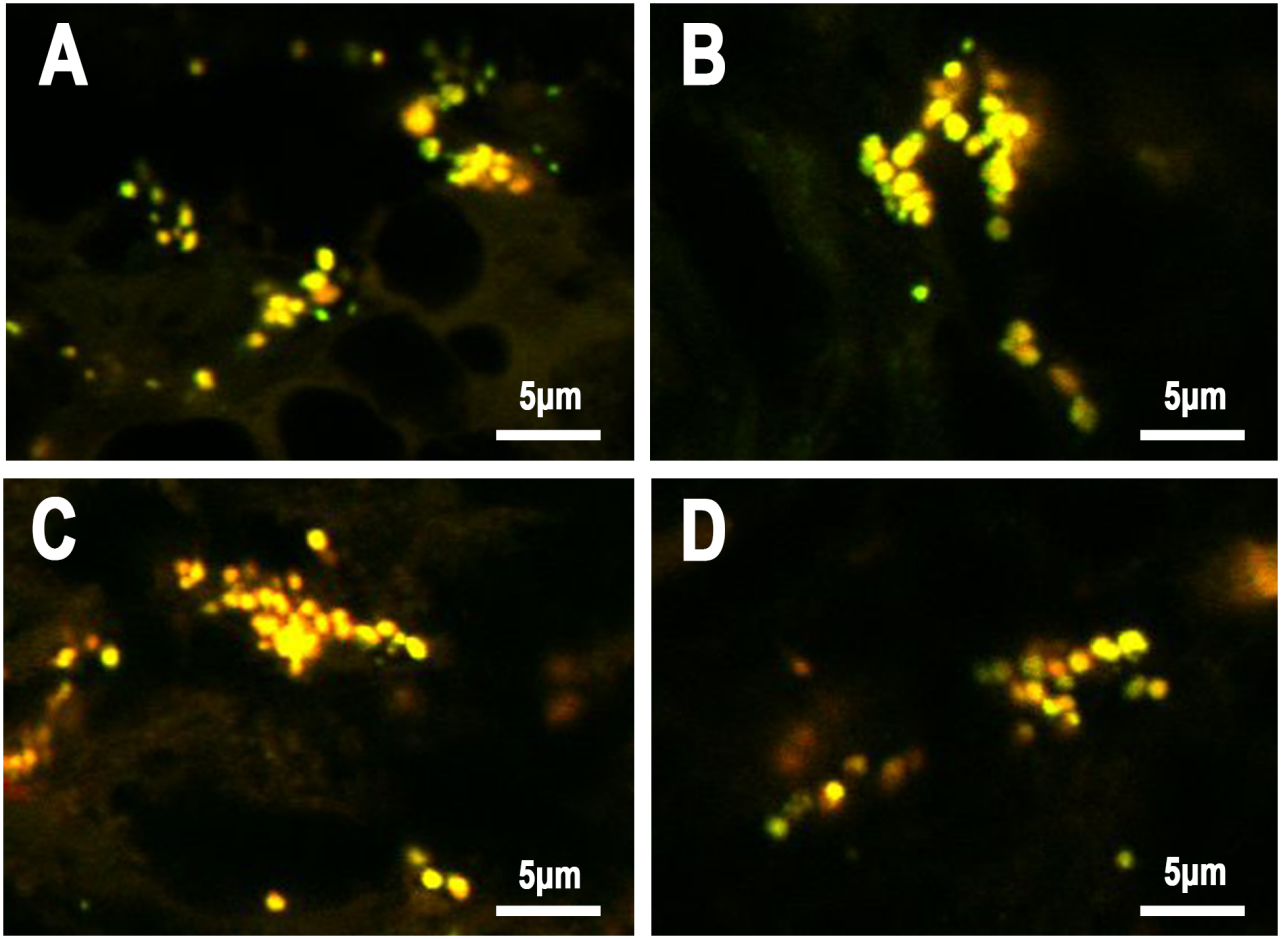
Co-localization of PAB-reactive PLTA antigen, IgA, IgM, and C3c detected by double fluorescence immunohistochemistry. A: Anti-IgA antibody (red) vs PAB antibody (green) after MT treatment, B: anti-IgM antibody (red) vs PAB antibody (green) after MT treatment, C: anti-IgM antibody (red) vs anti-IgA antibody (green), and D: anti-C3c antibody (red) vs PAB antibody (green) after MT treatment. Many PAB-reactive SRBs were also positive for IgA, IgM, and C3c, showing yellow-colored double-positive signals (A, B, and D, respectively). Both IgA and IgM colocalized with these SRBs, indicated by yellow-colored double-positive signals (C).

Double fluorescence IHC was performed to evaluate immunoglobulins and complement bound to PAB-reactive SRBs (Fig 8). Many of the PAB-reactive SRBs in sinus macrophages were positive for immunoglobulins (mainly IgA and IgM), and a few of them were also positive for complement. These immunoglobulin-bound SRBs were almost all positive for both anti-IgA and anti-IgM antibodies. A few of the PAB-reactive SRBs in sinus macrophages were negative for immunoglobulins.

**Fig 9:**
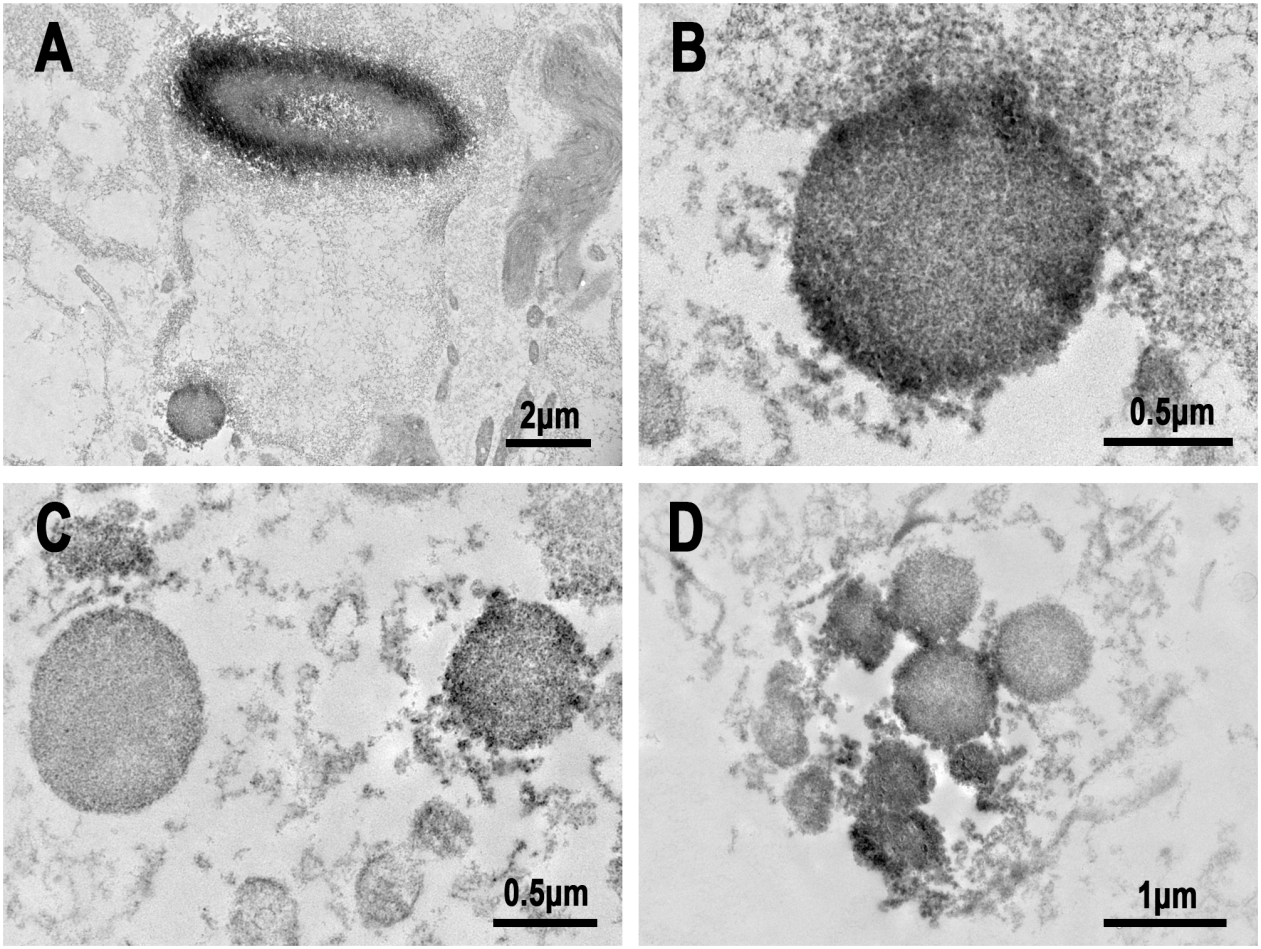
Immuno-electron microscopy images of SRBs in sinus macrophages of sarcoid lymph nodes. A and B: IHC with PAB antibody after MT treatment, C: IHC with anti-IgA antibody, and D: IHC with anti-IgM antibody. Note that dense black-colored reaction products by each antibody were located along the peripheral rim of the SRBs. A similar distribution of PAB-reactivity was observed in a large spherical-shaped HW body (A).

Immuno-electron-microscopic examination was performed to locate the immunoglobulins and PLTA of SRBs in sinus macrophages of sarcoid lymph nodes (Fig 9). PAB-reactive antigen PLTA located at the peripheral rim of SRBs as well as in HW bodies. Immunoglobulins (IgA and IgM) were also located mainly at the peripheral rim of SRBs.

### Electron-microscopy observation of SRBs

**Fig 10:**
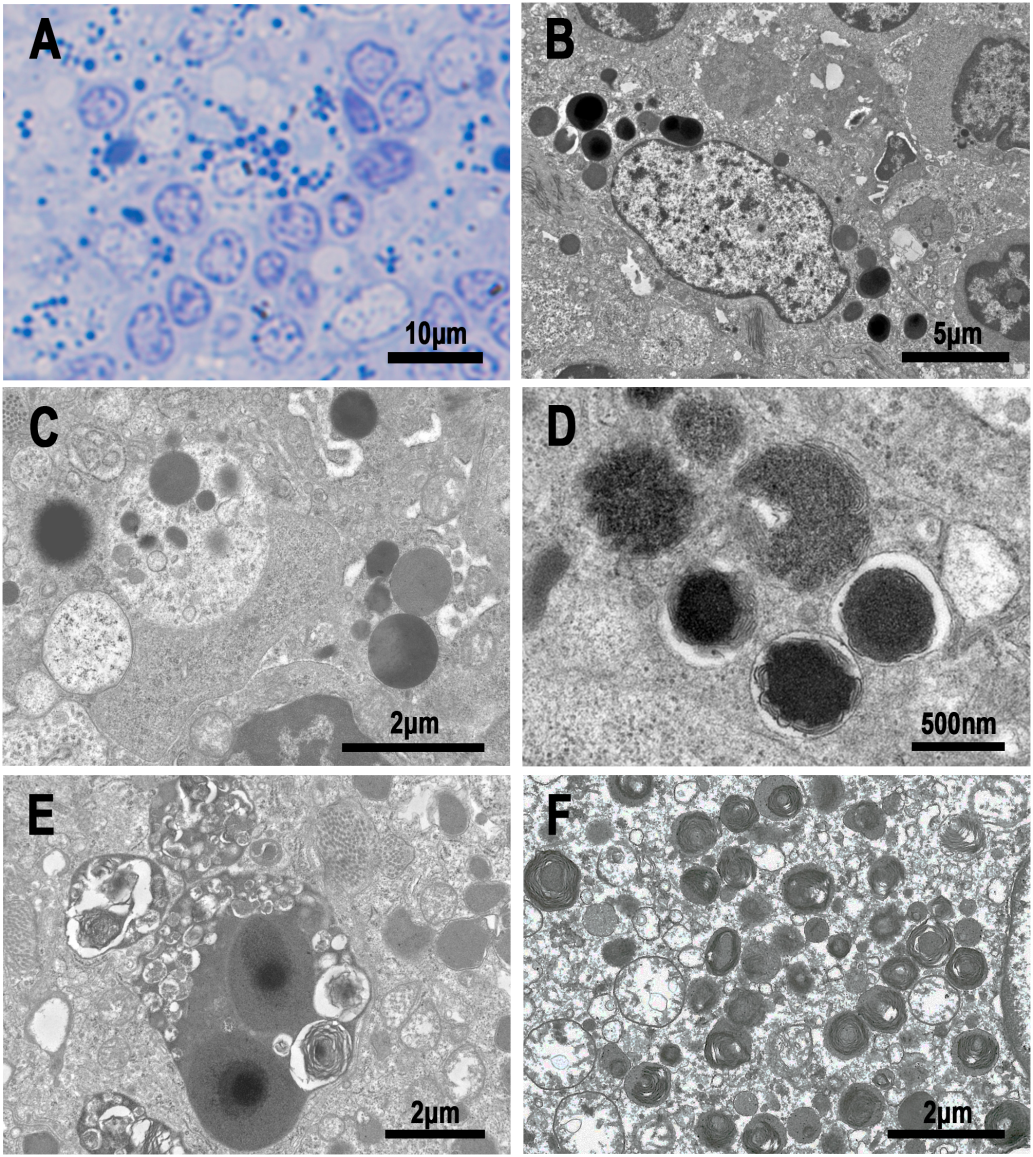
Electron-microscopy images of small round bodies and HW bodies in sinus macrophages of sarcoid lymph nodes. A: Light-microscopy image of an Epon-embedded section stained with methylene blue. Note the many SRBs and a few round or spherical-shaped HW bodies. B: Transitional forms between SRBs and round-shaped HW bodies. C: A phagolysosome containing SRBs. D: Phagolysosomes containing denatured SRBs with peripheral lamellar formation. E: A large phagolysosome containing HW bodies and many lamellar bodies. F: Many denatured SRBs observed in an epithelioid cell of sarcoid granuloma.

Two representative cases of sarcoid lymph nodes containing many IIC-forming *P*. *acnes* were subjected to conventional electron-microscopy examination (Fig 10). HW bodies were easily identified in sinus macrophages based on their size (3–5 μm in diameter) and a round or ovoid-shaped structure with a central dense core. Small round-shaped electron-dense bodies (0.5–2 μm in diameter) surrounded by a limiting membrane were detected in sinus macrophages and were especially abundant in sinus macrophages with HW bodies. Transitional forms between HW bodies and SRBs were occasionally observed. Many SRBs and a few HW bodies were occasionally found in phagolysosomes containing degenerated or lamellar bodies.

## Discussion

In the present study, we found IICs bound to immunoglobulins (mainly IgA and IgM) in many sarcoid lymph node samples. These IICs were located in sinus macrophages and could be easily differentiated morphologically from immunoglobulin-producing plasma cells. Some of these IICs also bound to complement (C1q or C3c), further suggesting that they are opsonized IICs and phagocytosed by sinus macrophages. IHC with MT treatment, which was developed in the present study to detect the IIC core antigen, revealed that these IICs were positive for PAB antibody. The location of PAB-reactive antigen and immunoglobulins or complement was identical in many sarcoid samples, which suggests that these IICs are indeed IIC-forming *P*. *acnes*.

The PAB antibody, which recognizes PLTA, is specific for *P*. *acnes* and does not react with other cutaneous propionibacteria such as *P*. *granulosum* and *P*. *avidum* [6]. MT treatment to expose the core PLTA antigen of IIC-forming *P*. *acnes* did not affect the specificity of the PAB antibody for *P*. *acnes*. Non-specific binding of the PAB antibody (IgM, κ) with bacterial proteins such as Protein A/G and secreted immunoglobulin binding protein from group A streptococcus [22] is unlikely, as no positive signal was observed with the LAM antibody (IgM, κ). Non-specific binding of the secondary antibody commonly used for detecting *P*. *acnes*, immunoglobulins, and complements to the sections also seems unlikely as no positive signal was detected by IHC without the primary antibody (PBS control). Moreover, the ISH results for *P*. *acnes* DNA confirmed the presence of *P*. *acnes* in sinus macrophages of sarcoid lymph nodes showing an identical distribution pattern to that of IIC-forming *P*. *acnes* detected by IHC with MT treatment.

The IIC-forming *P*. *acnes* seem to have been formed extracellularly by an in situ antigen-antibody reaction between the PAB-reactive PLTA antigen of *P*. *acnes* cells and the plasma anti-PLTA antibody. Basically, granulomatous reactions are cellular responses to irritating, persistent, and poorly soluble substances. Antibody-antigen complexes may provide a stimulus for granulomatous inflammation if the complex is insoluble and relatively indigestible [19]. According to these basic principles of granuloma formation, the frequent and abundant detection of IIC-forming *P*. *acnes* in sinus macrophages of sarcoid lymph nodes provides support for an etiologic link between sarcoidosis and *P*. *acnes*.

Negi et al [6] reported that many PAB-reactive SRBs are detected by IHC with the PAB antibody in sarcoid granuloma cells and paracortical macrophages, but not in macrophages of the lymphatic sinus where they noted only PAB-reactive HW bodies. The failure to detect PAB-reactive SRBs in sinus macrophages in the previous study may be due to the presence of immune complexes formed by PAB-reactive SRBs. The disturbed immunoreactivity of the PAB antibody with IIC-forming *P*. *acnes* in tissue sections without MT treatment is likely due to epitope competition [23] between the PAB antibody and the plasma anti-PLTA antibody.

Anti-PLTA antibody is a major infection antibody specific to *P*. *acnes* that is commonly induced in healthy adult subjects by the commensal bacterium, although many other *P*. *acnes* antigens also induce anti-*P*. *acnes* antibodies [8,10]. Indeed, anti-PLTA antibody was detected in all plasma samples used for the blocking experiments in our study. On PLTA-coated ELISA plates, the reactivity of the PAB antibody with PLTA was inhibited by previous incubation of the plates with human plasma samples, accompanied by simultaneous binding of plasma immunoglobulins (IgG > IgA > IgM) and complement (C1q > C3c). Similarly, on tissue sections of *P*. *acnes*-infected rat liver, PAB-reactivity with *P*. *acnes* cells in Kupffer cells was totally abolished by previous incubation of the sections with human plasma samples. Moreover, the ability of human plasma samples to block PAB-reactivity was also observed for the immunoglobulin or IgG fractions of a plasma sample. These results from the blocking experiments strongly suggest that plasma anti-PLTA antibodies contribute to the blocking effect against PAB-reactivity via an epitope competition for their binding to PLTA located at the peripheral margin of *P*. *acnes*. These findings also suggest that with IHC without prior MT treatment, the IIC-forming *P*. *acnes* in sinus macrophages of the lymph nodes are barely detected by the PAB antibody.

The MT treatment (microwaving followed by a brief trypsin digestion) was developed in the present study to detect the PAB-reactive core antigen of IIC-forming *P*. *acnes*. MT treatment seems to dissociate the antigen-antibody binding between PLTA and immunoglobulins of IIC-forming *P*. *acnes* and allow the PAB antibody to react with the exposed PLTA antigen that is resistant to trypsin digestion. This assumption is supported by the results of the blocking experiment in which PAB-reactivity with IIC-forming *P*. *acnes* that had been recovered by MT treatment was abolished by incubation with human plasma samples before the primary antibody reaction with the PAB antibody.

The detection of IIC-forming *P*. *acnes* in sinus macrophages suggests that these *P*. *acnes* were exposed to the extracellular space before being phagocytosed. One possibility is that these *P*. *acnes*-derived IICs originally formed outside the lymph node in organs located upstream of the lymphatic flow. Predominant detection of IgA- and IgM-bound *P*. *acnes* may support this possibility, whereas there was no correlation detected between the location of the lymph node biopsy and the IHC detection status of these IIC-forming *P*. *acnes* therein. Another possibility is that they were formed in the lymph nodes. Negi et al [6] suggested that *P*. *acnes* proliferates intracellularly in paracortical macrophages of lymph nodes. Some macrophages with intracellular proliferation of *P*. *acnes* may be disrupted without any direct contribution to granuloma formation and extracellular *P*. *acnes* are locally phagocytosed in sinus macrophages just after IIC formation with anti-PLTA antibodies in stromal lymphatic fluid. Multivalent IgA and IgM IICs may be more susceptible to local phagocytosis than monovalent IgG IICs, because the frequency and number of IgG IICs was far lower than those of IgA and IgM IIC in sarcoid lymph nodes. The IgG IICs may have escaped local phagocytosis and behaved as circulating immune complexes in sarcoidosis patients.

IIC formation is a physiologic host defense reaction. IgA and IgM IICs were also found in 21% and 29% of control lymph nodes, respectively, where MT treatment did not work efficiently, producing no prominent increase in the number or frequency of PAB-reactive SRBs. Furthermore, the IIC particles in control lymph nodes were generally smaller in size than those observed in sarcoid samples. These observations suggest that most of the IICs detected in control lymph nodes were formed with antigens other than *P*. *acnes*.

Although PAB-reactive SRBs (*P*. *acnes*) appear to be more frequent and abundant in sarcoidosis tissues, they are also observed in control tissues in the absence of granulomatous inflammation. These results are consistent with those obtained by bacterial culture [3,24,25] and quantitative polymerase chain reaction [4,26,27]. A few *P*. *acnes* are found in 20% of non-sarcoid lymph nodes by bacterial culture [3], 15% of non-sarcoid lymph nodes by quantitative polymerase chain reaction [4], and 22% of non-sarcoid lymph node samples by immunohistochemistry [6]. According to the etiology of sarcoidosis as an allergic endogenous infection [1], occasional detection of a few *P*. *acnes* in non-granulomatous areas of the lymph nodes from non-sarcoid patients suggests that latent *P*. *acnes* infection occurs even in healthy people [28]. Alternatively, another possible interpretation for the occasional presence of intracellular *P*. *acnes* in control lymph nodes is that *P*. *acnes* are simply collected by macrophages in both control and sarcoidosis tissues, and that this characteristic is enhanced by the presence of granulomas due to the specialized function of these macrophage-laden structures [29]. Further studies are needed to address the role of *P*. *acnes* in the etiology of sarcoidosis.

The difference between the sarcoid and control tissues seems to be the predominant presence of either immunoglobulin-free or immunoglobulin-bound *P*. *acnes* in sinus macrophages. Immunoglobulin-free *P*. *acnes* in sinus macrophages, including HW bodies, which can be detected without MT treatment, seems to be intracellularly persistent latent *P*. *acnes*. In contrast, immunoglobulin-bound *P*. *acnes* (IIC-forming *P*. *acnes*), which can be detected with MT treatment, seems to be formed after recent bacterial proliferation due to endogenous reactivation of latent *P*. *acnes*. According to this assumption, the results of the present study suggest that endogenous activation of latent *P*. *acnes* occurs in most sarcoid lymph nodes, some control lymph nodes with lymphadenitis, and in no control lymph nodes without inflammation. Endogenous activation of latent *P*. *acnes* may trigger the onset of sarcoid granuloma formation only in susceptible subjects (e.g., sarcoidosis patients) with Th1 hypersensitivity to this commensal bacterium [7–11,30].

Most of the HW bodies were negative for immunoglobulins, and thus the detection frequency of HW bodies was not increased by MT treatment to detect IIC-forming *P*. *acnes*. Negi et al [6] suggested that HW bodies are cell wall-deficient *P*. *acnes* that are persistent in sinus macrophages, which was supported by the observation of the cell wall-deficient bacterial structure of HW bodies, unusual dividing feature of the bodies, and SRBs budding from the HW bodies. In the present study, we also observed electron-dense SRBs in sinus macrophages, many of which were PAB-reactive and colocalized with IgA and IgM immunoglobulins. Some of these SRBs were occasionally observed in phagolysosomes with a degraded appearance and lamellar body formation. PAB-reactive HW bodies were closely associated in their morphology and distribution with PAB-reactive SRBs. Michailova et al [31] used electron microscopy to report that the drug-resistant *M*. *tuberculosis* L-form exhibits variability in size and morphology. The electron-microscopy features of PAB-reactive SRBs were consistent with those observed by Michailova et al [31,32] in terms of the size and morphology. Small-sized L-form bacteria are thought to indicate an infective phase [33]. Although immune complex formation against an infective form of *P*. *acnes* may be useful for preventing systemic infection, phagocytosis of IIC-forming *P*. *acnes* may cause persistent infection by the subsequent formation of HW bodies in sinus macrophages.

The detection frequency by IHC of *P*. *acnes* within granulomas was not very different with and without MT treatment (95% vs 92%). We detected more PAB-reactive SRBs in granuloma cells after MT treatment compared to without MT treatment, however, in some samples from sarcoidosis patients. This observation suggests that sarcoidosis granuloma formation is caused not only by macrophages with intracellular proliferation of *P*. *acnes*, but also by macrophages that phagocytosed IIC-forming *P*. *acnes*. PAB-reactivity of *P*. *acnes* detected by IHC with MT pretreatment was generally weaker in granuloma cells than in sinus macrophages, which suggests that granuloma cells have a greater capacity for intracellular digestion than macrophages [6,34,35].

The present study has several limitations. First, we could not discriminate between immunoglobulin-free *P*. *acnes* and IIC-forming *P*. *acnes* by enzyme IHC with the PAB antibody after MT treatment, whereas we could estimate the frequency and amount of IIC-forming *P*. *acnes* in sinus macrophages by IHC with PAB antibody after MT treatment because without MT treatment there were generally few or no immunoglobulin-free *P*. *acnes* other than HW bodies in sinus macrophages. Second, immuno-electron microscopy was performed with FFPE tissue samples, and thus we could not determine the detailed structure of the PAB-reactive SRBs. We compared the results of the immuno-electron and conventional electron microscopy to identify the IIC-forming *P*. *acnes*. For further analysis of IIC-forming *P*. *acnes*, a more detailed electron microscopic examination should be performed using an immunogold labeling method with fresh tissue samples that have been appropriately-fixed for this purpose.

In conclusion, abundant *P*. *acnes*-derived IICs bound to IgA and IgM were detected in sinus macrophages of the lymph nodes from many sarcoidosis patients. These findings may reflect local proliferation of *P*. *acnes* in sarcoid lymph nodes. *P*. *acnes*-derived IICs may provide a stimulus for granuloma formation in the lymph nodes in susceptible patients with *P*. *acnes* hypersensitivity.

## Supporting information

S1 Table Comparison of clinical profiles between sarcoidosis and control patients. (DOCX)

S2 Table The specificity of PAB antibody for *P*. *acnes* examined by IHC with or without MT treatment. (DOCX)

S1 Fig IHC with or without MT treatment using LAM antibody or PBS instead of the PAB antibody. Identical areas of serial sections from a sarcoid lymph node containing many IICs in sinus macrophages (A-F) and from *P*. *acnes*-infected rat liver (G-L). A, D, G, and J: IHC with PAB antibody (IgM, κ), B, E, H, and K: LAM antibody (IgM, κ), and C, F, I, and L: PBS control. No positive signals were observed by IHC with or without MT treatment when the LAM antibody or PBS was used instead of the PAB antibody. Scale bar: 20 μm.
(TIF)

S2 Fig In situ hybridization to detect *P*. *acnes* DNA. In situ hybridization (ISH) using catalyzed reporter deposition for signal amplification with digoxigenin-labeled oligonucleotide probe that complemented the 16S rRNA of *P*. *acnes* was performed with sarcoid lymph node samples having many IIC-forming *P*. *acnes* in sinus macrophages. Positive ISH signals were observed in sarcoid granuloma cells (A) and in hyperplastic-sinus macrophages (B). The distribution pattern of these positive ISH signals for *P*. *acnes* DNA was almost identical to that of the IHC positive signals with the PAB antibody after MT treatment. Scale bar: 20 μm.
(TIF)

S3 Fig Increased PAB-reactivity after MT treatment in sarcoid granulomas. In a representative case of a sarcoid lymph node in which PAB-reactivity was increased in a granuloma, identical areas of the granuloma are shown in semi-serial sections; HE stain (A), IHC with anti-human IgA antibody (B), IHC with PAB antibody (C), and IHC with PAB antibody after MT treatment (D). In the granuloma, the number of PAB-reactive SRBs is increased in the section with MT treatment (D) compared with the section without MT treatment (C). Scale bar: 20 μm.
(TIF)
