# Supplementary material for: Propionibacterium acnes-derived insoluble immune complexes in sinus macrophages of lymph nodes affected by sarcoidosis
Source: PLoS One. 2018 Feb 5;13(2):e0192408. doi: 10.1371/journal.pone.0192408 (PMC5798840; doi:10.1371/journal.pone.0192408)
Supplement: S1 Table — (DOCX) [file pone.0192408.s001.docx]

S1 Table. Comparison of clinical profiles available between sarcoidosis and control patients.

|  | **Sarcoidosis** | **Control**  **total** | **Control** | | | |
| --- | --- | --- | --- | --- | --- | --- |
|  |  |  | **Colon**  **cancer** | **Gastric cancer** | **Necrotizing lymphadenitis** | **Reactive lymphadenitis** |
| Number | 38 | 90 | 16 | 19 | 27 | 28 |
| Subjects (men / Women) | 16 / 22 | 47 /43 | 11 / 5 | 14 / 5 | 9 / 18 | 13 / 15 |
| Age, years | 56.3 ± 16.2 | 49.1 ± 20.5 | 66.0 ± 11.7 ^*1^ | 67.9 ± 12.3 ^*2^ | 31.9 ± 12.0 ^*3^ | 43.2 ± 17.7 ^*4^ |
| Location of lymph node biopsy |  |  |  |  |  |  |
| Hilum | 9 | 1 |  |  |  | 1 |
| Mediastinal | 6 |  |  |  |  |  |
| Tracheal | 2 |  |  |  |  |  |
| Abdominal | 1 | 36 | 16 | 19 |  | 1 |
| Para arterial | 1 |  |  |  |  |  |
| Cervical | 8 | 42 |  |  | 25 | 17 |
| Inguinal | 4 | 5 |  |  |  | 5 |
| Supraclavicular | 4 | 1 |  |  |  | 1 |
| Submandibular |  | 1 |  |  |  | 1 |
| Axilla |  | 4 |  |  | 2 | 2 |
| Anterior scalen | 3 |  |  |  |  |  |
| Serum immunoglobulin |  |  |  |  |  |  |
| IgG (mg/dl) | 1484.1 ± 448.7 | 1536.0 ± 452.6 | NA | NA | 1548.3 ± 387.1 | 1519.7 ± 541.3 |
| IgA (mg/dl) | 293.5 ± 134.4 | 276.0 ± 113.9 | NA | NA | 245.9 ± 85.4 | 321.1 ± 139.5 |
| IgM (mg/dl) | 102.5 ± 45.9 | 139.3 ±87.4 | NA | NA | 166.4 ± 95.9 ^*5^ | 98.6 ± 54.5 |

*1; *P* = 0.0336, *2; *P* = 0.0118, *3; *P* < 0.0001, *4; *P* = 0.0016, *5; *P* = 0.0177 compared with sarcoidosis patients (Holm-Sidak's multiple comparisons test). NA: not available.
