# Supplementary material for: Propionibacterium acnes-derived insoluble immune complexes in sinus macrophages of lymph nodes affected by sarcoidosis
Source: PLoS One. 2018 Feb 5;13(2):e0192408. doi: 10.1371/journal.pone.0192408 (PMC5798840; doi:10.1371/journal.pone.0192408)
Supplement: S2 Table — (DOCX) [file pone.0192408.s002.docx]

S2 Table. The specificity of the PAB antibody to *P. acnes* in IHC with and without MT treatment.

| Bacteria | Strain | PAB-reactivity with the bacterium by IHC | |
| --- | --- | --- | --- |
|  |  | without MT treatment | with MT treatment |
| *Propionibacterium acnes* | ATCC 6919 | + | + |
| *Propionibacterium acnes* | ATCC 11828 | + | + |
| *Propionibacterium granulosum* | ATCC 25564 | - | - |
| *Propionibacterium avidum* | ATCC 25577 | - | - |
| *Propionibacterium propionicum* | ATCC 14157 | - | - |
| *Propionibacterium freudenreichii* | ATCC 6207 | - | - |
| *Propionibacterium jensenii* | ATCC 4868 | - | - |
| *Propionibacterium thoenii* | ATCC 4874 | - | - |
| *Propionibacterium acidipropionici* | ATCC 25562 | - | - |
| *Mycobacterium tuberculosis* | ATCC 33152 | - | - |
| *Bacteroides fragilis* | ATCC 25285 | - | - |
| *Fusobacterium nucleatum* | ATCC 25586 | - | - |
| *Listeria monocytogenes* | ATCC 15313 | - | - |
| *Staphylococcus aureus* | ATCC 25923 | - | - |
| *Streptococcus pneumoniae* | ATCC 33400 | - | - |
| *Helicobacter pylori* | ATCC 43504 | - | - |
| *Escherichia coli* | ATCC 11775 | - | - |
| *Enterococcus faecalis* | ATCC 19433 | - | - |
| *Pseudomonas aeruginosa* | ATCC 27853 | - | - |
| *Klebsiella pneumoniae* | ATCC 13884 | - | - |
