# Supplementary figures and images for: Propionibacterium acnes-derived insoluble immune complexes in sinus macrophages of lymph nodes affected by sarcoidosis
Source: PLoS One. 2018 Feb 5;13(2):e0192408. doi: 10.1371/journal.pone.0192408 (PMC5798840; doi:10.1371/journal.pone.0192408)

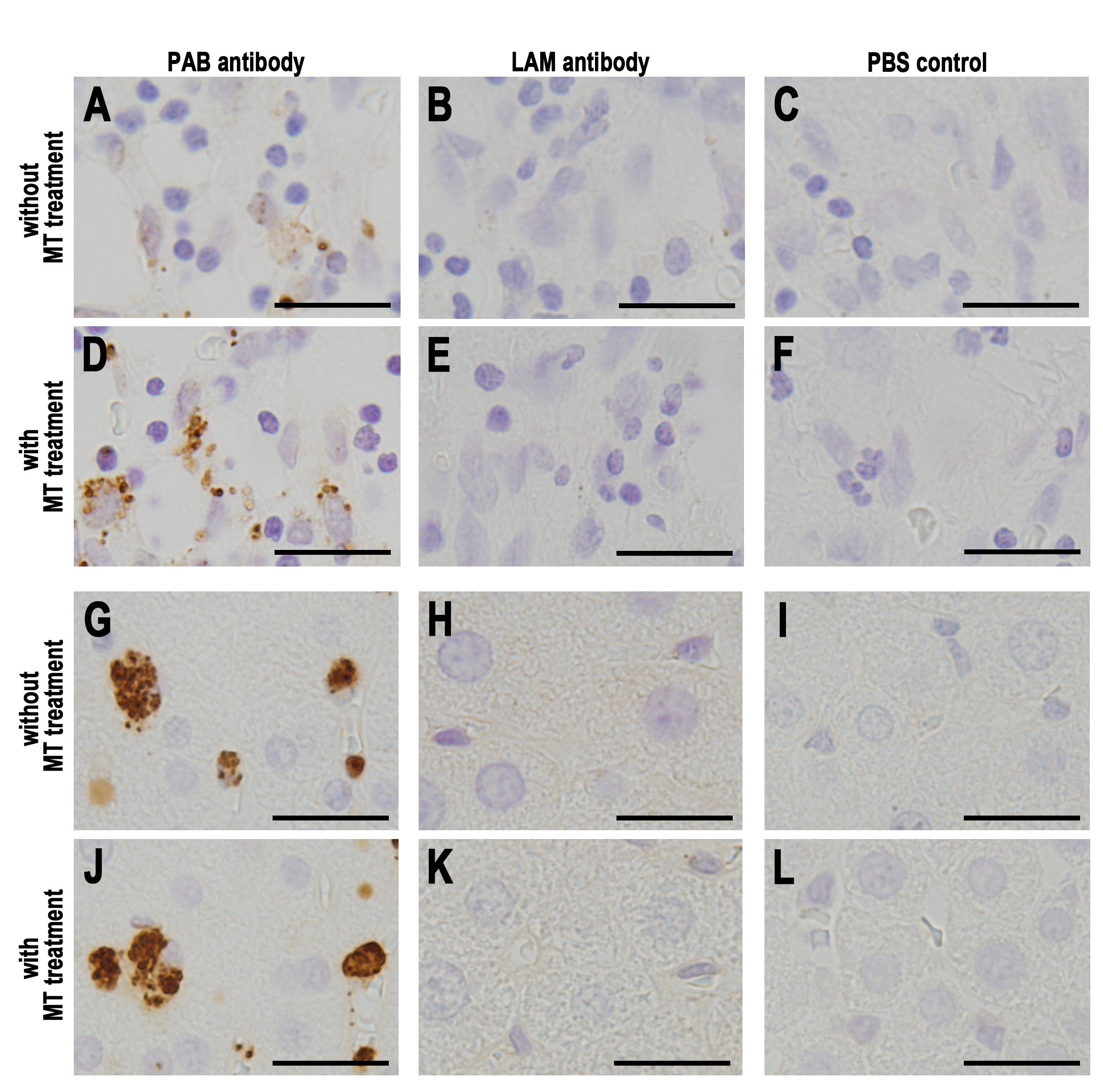

Supplement: S1 Fig — Identical areas of serial sections from a sarcoid lymph node containing many IICs in sinus macrophages (A-F) and from P. acnes-infected rat liver (G-L). A, D, G, and J: IHC with PAB antibody (IgM, κ), B, E, H, and K: LAM antibody (IgM, κ), and C, F, I, and L: PBS control. No positive signals were observed by IHC with or without MT treatment when the LAM antibody or PBS was used instead of the PAB antibody. Scale bar: 20 μm. (TIF) [file pone.0192408.s003.tif]

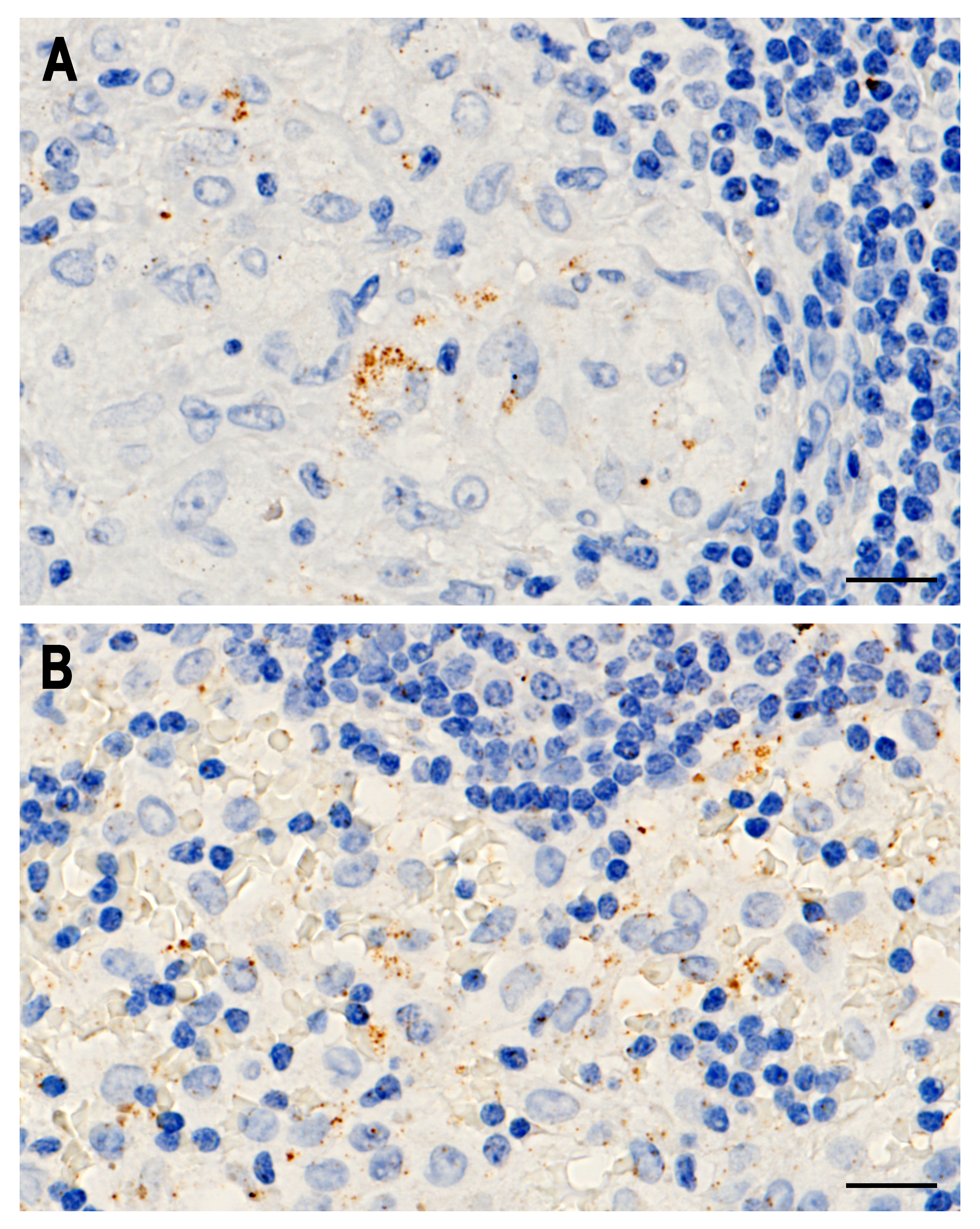

Supplement: S2 Fig — In situ hybridization (ISH) using catalyzed reporter deposition for signal amplification with digoxigenin-labeled oligonucleotide probe that complemented the 16S rRNA of P. acnes was performed with sarcoid lymph node samples having many IIC-forming P. acnes in sinus macrophages. Positive ISH signals were observed in sarcoid granuloma cells (A) and in hyperplastic-sinus macrophages (B). The distribution pattern of these positive ISH signals for P. acnes DNA was almost identical to that of the IHC positive signals with the PAB antibody after MT treatment. Scale bar: 20 μm. (TIF) [file pone.0192408.s004.tif]

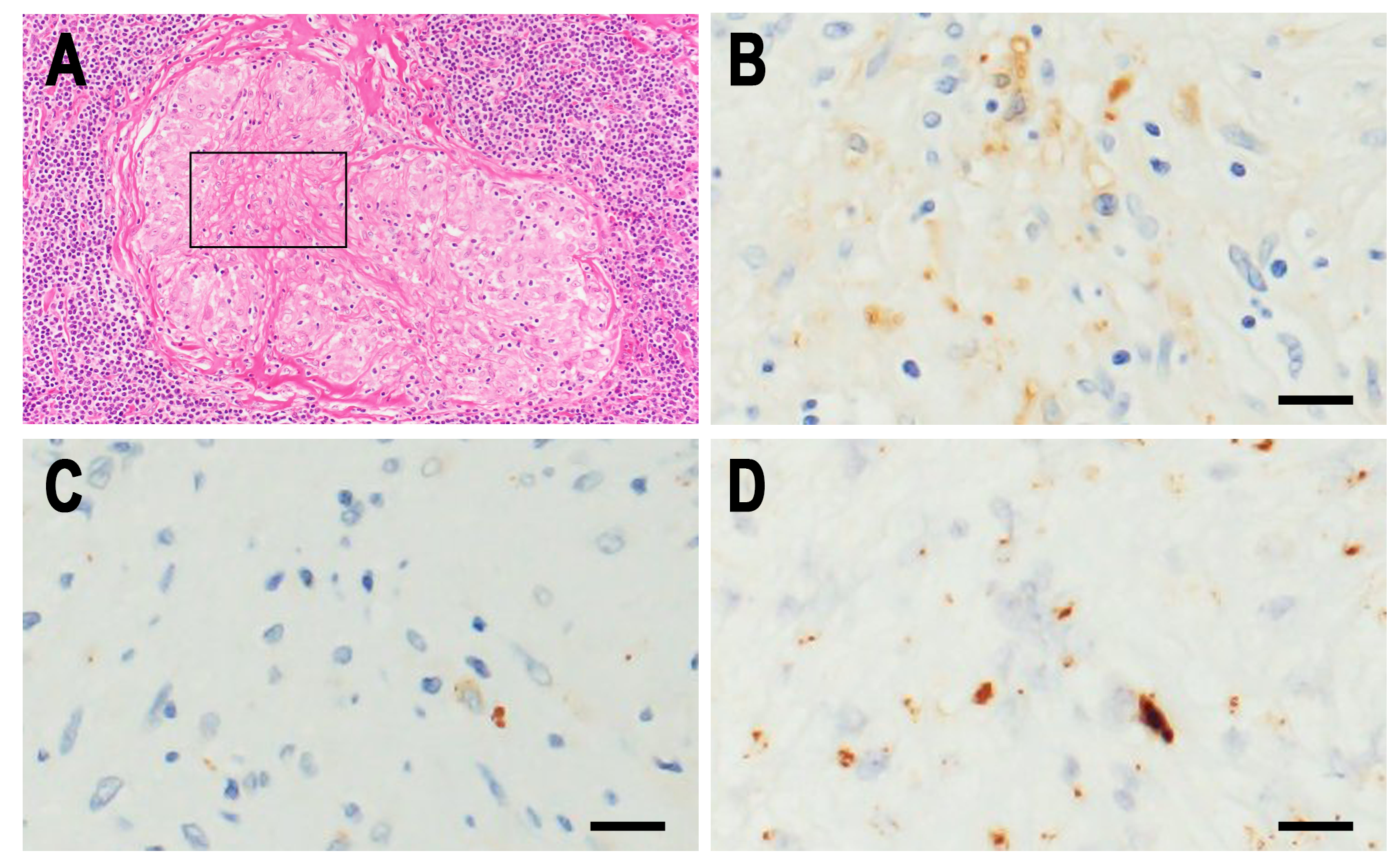

Supplement: S3 Fig — In a representative case of a sarcoid lymph node in which PAB-reactivity was increased in a granuloma, identical areas of the granuloma are shown in semi-serial sections; HE stain (A), IHC with anti-human IgA antibody (B), IHC with PAB antibody (C), and IHC with PAB antibody after MT treatment (D). In the granuloma, the number of PAB-reactive SRBs is increased in the section with MT treatment (D) compared with the section without MT treatment (C). Scale bar: 20 μm. (TIF) [file pone.0192408.s005.tif]
